# Rare but overlooked: choroidal metastasis as a late manifestation of colon cancer with review of literature

**DOI:** 10.1093/omcr/omaf175

**Published:** 2025-10-29

**Authors:** Poorva Vias, Ashish Saklani, Mayur Garkar, Gaurav Sharma, Vandana Thakur, Pratibha Prashar, Brish Bhanu Bhardwaj

**Affiliations:** Department of Radiation Oncology, Dr. Rajendra Prasad Government Medical College, Kangra at Tanda, Himachal Pradesh 176001, India; Department of Radiodiagnosis, Dr. Rajendra Prasad Government Medical College, Kangra at Tanda, Himachal Pradesh 176001, India; Department of Ophthalmology, Dr. Rajendra Prasad Government Medical College, Kangra at Tanda, Himachal Pradesh 176001, India; Department of Ophthalmology, Dr. Rajendra Prasad Government Medical College, Kangra at Tanda, Himachal Pradesh 176001, India; Department of Radiation Oncology, Dr. Rajendra Prasad Government Medical College, Kangra at Tanda, Himachal Pradesh 176001, India; Department of Radiation Oncology, Dr. Rajendra Prasad Government Medical College, Kangra at Tanda, Himachal Pradesh 176001, India; Department of Pathology, Dr. Rajendra Prasad Government Medical College, Kangra at Tanda, Himachal Pradesh 176001, India

**Keywords:** colorectal cancer, choroidal metastasis, ocular metastasis, diminished vision, systemic chemotherapy

## Abstract

Choroidal metastasis is a rare but significant manifestation of systemic malignancies, most commonly originating from breast and lung cancers. Metastatic spread to the choroid from colorectal cancer (CRC) is exceedingly uncommon, with limited cases reported in the literature. However, it poses a significant diagnostic and therapeutic challenge when it occurs. 33 years old female with Adenocarcinoma of the colon T3N0M0 initially treated curatively, developed lung metastasis 18 months later. At 23 months post-diagnosis, she presented with blurred vision and severe pain. Ophthalmologic exam and imaging (OCT, ultrasonography, MRI) confirmed a choroidal lesion with retinal detatchment. A comprehensive review of available case reports and series of choroidal metastases originating from colorectal cancer was conducted. Data on patient demographics, symptoms, diagnostic modalities, treatment options, and outcomes were analyzed. Choroidal metastasis from CRC serves as a red flag for extensive disease and the most common presenting symptom is visual impairment, including blurred vision, scotomas, or diplopia. Clinically significant implications of this case are indication of advanced systemic spread, need for prompt multidisciplinary evaluation through clinical examination, fundoscopic findings, and imaging modalities such as optical coherence tomography (OCT), ultrasonography, and magnetic resonance imaging (MRI) andtreatment options which include external beam radiotherapy (EBRT), systemic chemotherapy, and targeted therapies to control ocular symptoms and improve visual function. Early recognition allows for integrated systemic and ocular care, and targeted local therapy can meaningfully improve visual outcomes.

## Introduction

Metastatic colon cancers are seen in 20% of patients and approximately 25–30% will eventually develop metastases over time [1].The most common sites of metastasis from colon cancer are liver, peritoneum followed by lungs [2].

Choroidal metastasis, although uncommon, represents the most frequent intraocular malignancy in adults, typically originating from systemic malignancies. It occurs when tumour cells intravasate into the bloodstream—facilitated by processes like epithelial-to-mesenchymal transition (EMT) and matrix metalloproteinase (MMP)–mediated extracellular matrix breakdown and circulate until they lodge in the richly vascularized posterior choroid via the posterior ciliary arteries [3]. Among all cancers, breast cancer in women and lung cancer in men are the most common primary sources of choroidal metastasis [4].

However, ocular metastases, specifically to the choroid, are exceedingly rare in CRC, with an incidence estimated to be 1 to 4% among patients with metastatic disease. This rarity is attributed to the ocular microenvironment and its immune-privileged status, which may limit metastatic colonization [4].

Choroidal metastases often present with nonspecific symptoms such as blurred vision, scotomas, or even asymptomatic findings on routine eye examinations. The diagnosis is typically established based on clinical history, ophthalmoscopy, imaging techniques (such as ultrasonography, fluorescein angiography, or optical magnetic resonance imaging), and occasionally biopsy. Identifying the primary tumor site is crucial, as the management of choroidal metastases largely depends on systemic therapy directed at the underlying malignancy.

Here, we present a case of young female presenting with metastatic colon cancer developing choroidal metastasis.

## Case report

**Figure 1:**
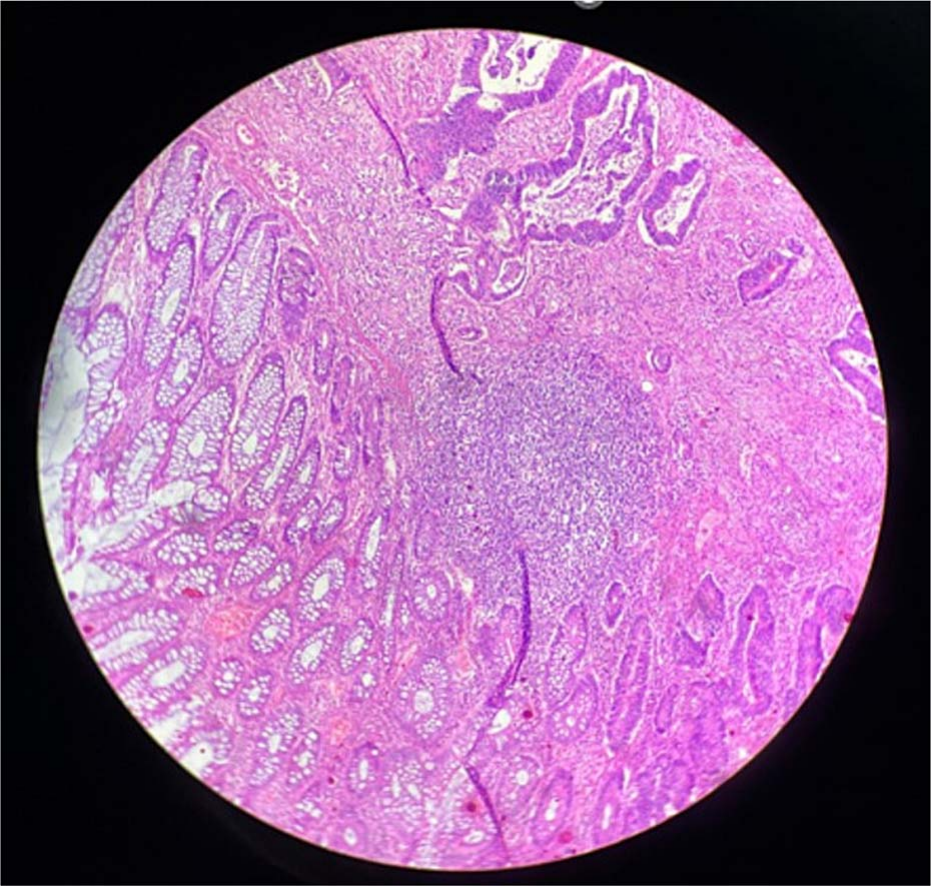
Histopathological section (10×) revealing arrangement in glandular pattern having moderate pleomorphism along with normal crypts and lymphoid follicles suggestive of adenocarcinoma.

A Thirty-three years old female with adenocarcinoma of sigmoid colon (Fig. 1) T3N0M0 (as per AJCC 8^th^ edition) underwent left hemicolectomy in January 2022 followed by six months of chemotherapy based on CAPOX regimen.

After one year of follow-up, patient developed shortness of breath which on PET-CT revealed pulmonary nodules in bilateral upper and lower lobes with largest nodule measuring 2.9×2.3 cm in right upper lobe abutting right mainstem bronchus with few enlarged right lower paratracheal and hilar lymph nodes, suggesting disease progression. Patient was started on second line chemotherapy based on CAPIRI plus Inj. Bevacizumab (Tab Capecitabine: 800 mg/m^2^ orally twice daily on Days 1–14, Inj. Irinotecan: 200 mg/m^2^ IV on Day 1, Inj Bevacizumab: 7.5 mg/kg IV on Day 1, every 3 weekly). Post 23 months of diagnosis after 7 cycles of chemotherapy, patient started having diminished vision and severe pain in left eye affecting her day to day activities.

**Figure 2:**
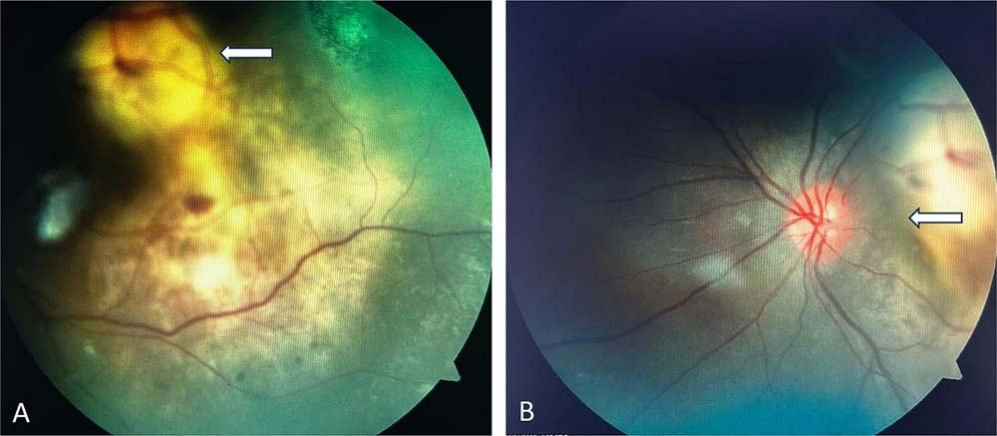
Indirect Ophthalmoscopic examination showing (A) Supratemporal choroidal mass suggestive of choroidal mass leading to (B) exudative retinal detachment.

On ophthalmic examination (Fig. 2) left eye visual acuity was hand movement close to face, pupil showing relative afferent pupillary defect (RAPD). The slit lamp examination was normal. On indirect ophthalmoscopic examination, media was clear, optical disc was normal, hypopigmented irregular mass with overlying blood two-disc diameter away from optic disc and two-disc diameter in size. Large smooth hypopigmented area was seen involving posterior pole. The foveal reflex was dull with patchy proliferative area suggesting exudative retinal detachment.

**Figure 3:**
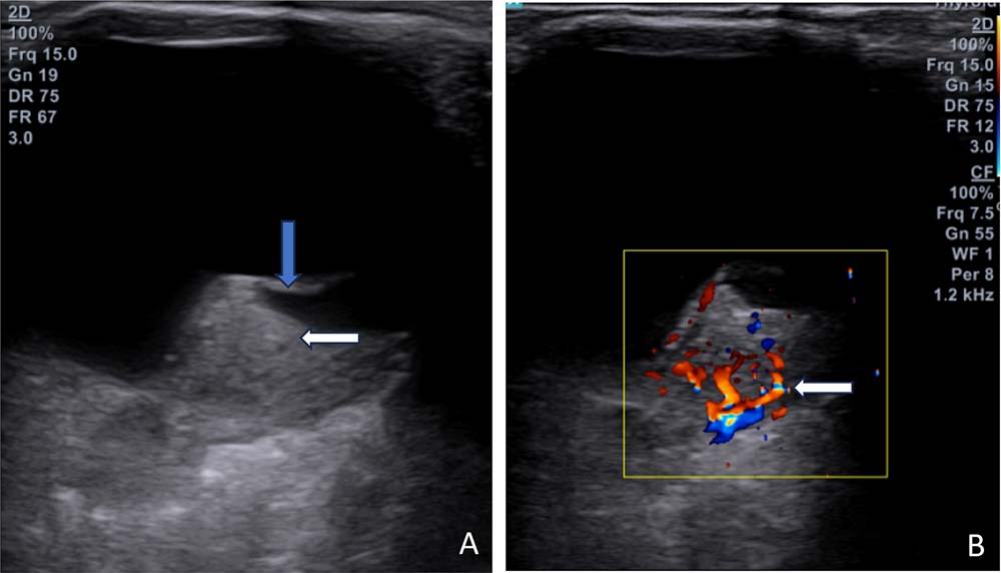
B-mode ultrasound scan of left orbit. (A): Sclerochoridal based echogenic soft tissue lesion (white arrow) in posterior segment overlying optic disc with adjacent retinal detachment (blue arrow) with subretinal fluid collection. (B): Color doppler ultrasound image shows intense vascularity (white arrow) in soft tissue lesion.

On B-Mode scan (Fig. 3) of left orbit an echogenic choroidal based soft tissue lesion measuring 6x8mm was seen in posterior segment overlapping optic disc. An echogenic membrane was seen overlying soft tissue lesion with underlying fluid suggesting retinal detachment. On colour doppler, intense vascularity was demonstrated suggestive of hypervascular lesion.

**Figure 4:**
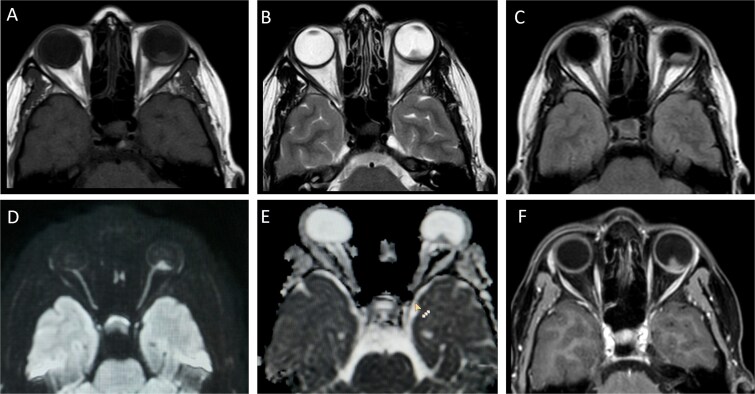
(A-C) T1, T2 and FLAIR images respectively show isointense soft tissue mass lesion in left orbit overlapping lateral aspect of optic disc. (D and E) diffusion and ADC map images shows diffusion restriction in left orbit lesion. (F) T1 fat-saturation post contrast image shows intense enhancement in orbital lesion.

On MRI Orbit, (Fig. 4) A well-defined soft tissue lesion measuring 5.8 x 7.8 mm was seen in temporal aspect of posterior segment of left eye. The lesion was found to be choroid based causing mild indentation and displacement of retina. Lesion was partially overlapping optic disc causing minimal medial displacement of optic nerve; however, no intrinsic abnormality or abnormal enhancement was seen in optic nerve. The lesion was found to be heterogeneously isointense on T1 and T2 weighted images. On Diffusion weighted images, diffusion restriction with corresponding low ADC (Apparent diffusion coefficient) values was seen indicating high cellularity and malignant nature. On T1 fat saturation post contrast images, enhancement was seen in lesion indicating rich vascularity. No scleral, retrobulbar or periocular extension seen. Patient did not want to getfurther investigations and targeted treatment thus only symptomatic treatment with Tab morphine 10 mg 4 hourly with CAPIRI+ Bevacizumab was given and last follow up was seen 3 months after the diagnosis of choroidal metastasis where pain was relieved but visual impairment was there and systemic disease persisted.

## Discussion

Metastatic ocular tumours are more common than primary ocular malignancies and choroid is the most common site of malignancies susceptible to hematogenous dissemination due to high vascularity [5]. The most common source of choroidal metastasis are from breast (40–47%) and lung (21–29%), whereas gastrointestinal cancers accounting for less than 4% [4, 6].

Colorectal malignancies metastasizing to the choroid is a rare event which is usually seen with metastasis to other sites also. In our case, colorectal malignancy was seen with metastasis to lung and choroid.

On detailed literature review to identify similar cases of choroidal metastasis from colorectal cancers, twenty-five cases were retrieved. Brief summary and comparison of all the cases is represented in Table 1 [5, 7–28].

**Table 1:** Summary of literature review of colorectal cancer patients with choroidal metastasis.

Author	Year	Age	Sex	TIME from initial diagnosis in months	Primary site	Other mets	Ocular symptom	Ocular site	Stage	Treatment	Outcome
KENNEDY et al [7]	1958	51	M	SYNCHRONOUS	RECTOSIGMOID	NO	BLURRED VISION	CHOROID, MACULA	NS	ENUCLEATION	9 M
HOWARD et al [8]	1968	63	M	36 M	COLON	NS	DIMINISHED VISION	CHOROID, RETINA, ORBIT	NS	ENUCLEATION	16 M
SCHNEIDER PA et al [9]	1978	NA	NA	NA	COLON	NA	NA	CHOROID	NS	RT	NA
COLE et al [10]	1985	48	F	23 M	RECTUM	LUNG	BLURRED VISION	CHOROID	T4A	CT,RT	4 M
TANO et al [11]	1989	30	M	SYNCHRONOUS	RECTUM	BONE, SKIN	BLURRED VISION	CHOROID	NS	ENUCLEATION	4 M
SHINJYO et al [12]	1989	78	M	7 M	COLON	SKIN	VISION LOSS	CHOROID	NS	NONE	9 M
WARD SD et al [13]	2000	52	F	SYNCHRONOUS	COLON	INTRA-ABDOMINAL	DIMINISHED VISION	CHOROID	NS	NONE	1 M
NAKAMURA et al [14]	2002	79	M	18 M	COLON	LUNG	CLOUDY VISION	CHOROID	T4A	CRT	12 M ALIVE
LINARES P et al [15]	2004	47	M	SYNCHRONOUS	RECTUM	LIVER, LUNG	BLURRED VISION	CHOROID	TXNXM1	CT,RT	9 M
FUJIWARA et al [16]	2004	53	M	30 M	RECTUM	LUNG, LIVER, BONE	VISION LOSS	CHOROID	NS	CRT	1 M
HISHAM et al [17]	2006	32	M	10 M	RECTUM	BREAST, BONE	EYE PAIN	CHOROID	T4A	NONE	2 M
KUO IC et al [18]	2008	65	F	20 M	COLON	BRAIN	VISION LOSS	CHOROID	NS	IVT BEV	5 M ALIVE
SASHIYAMA et al [19]	2010	49	M	15 M	RECTUM	LUNG, BONE	VISION LOSS	CHOROID	T3	CT	11 M
LIN CJ et al [20]	2010	43	M	8 M	COLON	BONE	VISION LOSS	CHOROID	NS	CT, BEV	4 M
MIYAKE et al [21]	2012	74	M	SYNCHRONOUS	RECTUM	LIVER, LUNG	VISION LOSS	CHOROID	NS	CT	8 M
TEI M et al [5]	2014	60	M	30 M	RECTUM	LUNG	FLOATERS	CHOROID	T1NXMX	CT, RT	27 M
KHAWAJA MR et al [22]	2015	60	F	42 M	RECTUM	LUNG	FLASHES	CHOROID	T3N1 M0	CT,RT, BEV	31 M
BOSS JD et al [23]	2016	68	F	96 M	RECTUM	LUNG, CEREBELLUM	FLASHES, FLOATERS	CHOROID	T3N0MX	INTRAVIT BEV	NS
HA JY et al [2]	2016	78	F	18 M	COLON	LUNG, SKIN, BRAIN	VISUAL DISTURBANCE	CHOROID	NS	CT	8 M
CRUZADO-SANCHEZ D et al [24]	2020	64	M	36 M	COLORECTUM	LUNG	EYE PAIN, VISION LOSS	CHOROID	T3N1 M0	ENUCLEATION	6 M
BICCAS NETO L et al [25]	2020	70	M	8 M	COLON	LIVER, LUNG	DIMINISHED VISION	CHOROID	NS	CT, PHOTODYNAMIC THERAPY	1 M ALIVE
AMISHA F et al [26]	2021	44	M	SYNCHRONOUS	RECTUM	LIVER, LUNG	EYE PAIN, VISION LOSS	CHOROID	NS	CT,RT	NS
DEMIR H et al [27]	2024	70	M	22 M	RECTUM	LUNG, BONE, ADRENAL	BLURRED VISION	CHOROID	T3N3 M0	RT	3 M
LAHHAM EE et al^28s^	2024	58	M	30 M	RECTUM	LIVER, LUNG	DIMINISHED VISION	CHOROID	T3N0MX	CT, RT, SYS BEV	5 M ALIVE
Present study	2024	33	F	18 M	COLON	LUNG	DIMINISHED VISION, PAIN	CHOROID	T3N0M0	CT, SYS BEV	3 M

The patients with choroidal metastasis may present with diminution of vision, blurred vision, floaters, flashes, ocular pain or eye movement disorder for which they should consult an ophthalmologist. Our patient had severe ocular pain and diminished vision which was acute in onset which on ophthalmic examination and further investigations showed choroidal metastasis in a known case of colorectal carcinoma. The typical manifestation of choroidal metastasis include plateau or flat creamy white to pale yellow mass, often seen with collection of subretinal fluid or retinal detachment [29].

The diagnosis is made on the basis of Ophthalmological examination, ultrasonography, angiography, optical coherence tomography, computed tomography or MRI orbit. Tissue diagnosis is not preferred due to the hypervascularity of this structure.

Due to the rarity of cases, exact treatment could not be suggested, however, the treatment options encompass systemic chemotherapy, radiotherapy, intravitreal inj. Bevacizumab, photodynamic therapy, enucleation or observation [24]. Chemotherapeutic agents (e.g. FOLFOX/CAPOX, FOLFIRI/CAPIRI ± intravenous bevacizumab,) reduce systemic tumor load and may indirectly shrink choroidal lesions [10, 15, 19–21]. External Beam Radiotherapy (e.g. 30–40 Gy in 10–20 fractions) is the mainstay for local control of choroidal metastases and achieves tumor control and vision stabilization or improvement in ~ 80–86% of patients [9, 10, 14–16, 28]. Intravitreal Bevacizumab 4 mg injections have mixed responses in the literature [18, 23]. Cases report with photodynamic therapy improved vision and decreased subretinal fluid in CRC metastasis, and broader metastatic disease with ~ 76–78% tumor regression in other cancers [25]. Enucleation is indicated for painful, blind eye with mass effect, unresponsive to other treatments [7, 8].

The treatment choices for choroidal metastasis depend on various factors including the general condition of the patient, status of primary disease, other metastatic sites, life expectancy and visual function. Our patient received systemic chemotherapy based on CAPIRI plus intravenous Inj Bevacizumab but died after 4 months of diagnosis.

Choroidal metastasis carries a very poor prognosis, with a median duration from diagnosis to death of 6 months, ranging from 0.5 to 47 months [29].

## Conclusion

Early recognition of ocular symptoms in CRC patients can enable targeted interventions (e.g. radiotherapy, chemotherapy, targeted therapy). Prompt multidisciplinary management is essential to preserving vision and improving quality of life, but despite local treatment, prognosis remains poor, emphasizing the importance of systemic disease control.. Further research is warranted to improve understanding of the pathophysiology, risk factors, and optimal treatment strategies for this rare metastatic manifestation.
